# Sake Brewing and Bacteria Inhabiting Sake Breweries

**DOI:** 10.3389/fmicb.2021.602380

**Published:** 2021-03-04

**Authors:** Hiromi Nishida

**Affiliations:** Department of Biotechnology, Toyama Prefectural University, Imizu, Japan

**Keywords:** bacterial flora, *koji*, *kuratsuki*, *Hatsuzoe*, *moto*, *Kocuria* sp., sake brewing

## Introduction

Sake is a Japanese traditional fermented alcoholic drink. It is brewed using *koji* mold to convert the starch in rice into sugar, which is then converted into ethanol by sake yeast. Two eukaryotic microorganisms, *Aspergillus oryzae* and *Saccharomyces cerevisiae*, are used for sake brewing, leading to highly efficient ethanol fermentation. The final ethanol concentration is ~20%, which is higher than that of beer and wine. The use of technology in the sake brewing process is remarkably high and involves parallel double fermentation. In contrast, beer is brewed using malt to convert the starch in barley into sugar, which is then converted into ethanol by beer yeast. In winemaking, wine yeast converts sugar from grapes into ethanol. Beer and wine processes can include also serial yeast-based double fermentation. It is well-known that bacteria, mainly lactic acid bacteria, can have a role in the production process of beer (sour beer) and wine (malolactic fermentation) (Berbegal et al., 2019; De Roos et al., 2020; Dysvik et al., 2020; Virdis et al., 2020). On the opposite, several alcoholic beverages, such as sake, are considered fermented only by eukaryotic microorganisms.

Beer, sake, and wine originated long before the discovery of microorganisms. Thus, these alcoholic drinks were produced without understanding the mechanism of ethanol fermentation. Surprisingly, sake undergoes pasteurization, called *hiire*, in the process of sake storage, and it has been recorded as performed 300 years before Pasteur reported the pasteurization method. The high level of biotechnology involved in sake brewing has been maintained for a long time. Sake breweries are widely distributed in Japan, and there may be much more to learn from the sake brewing process. Sake has different grades depending on the degree of polishing of rice. The highest grade (*Daiginjo*) is produced using polished rice with 50% or more removal of the outer layer of the grain. In addition, sake is distinguished by the addition of distilled alcohol. Sake without distilled alcohol is called *Junmaishu*. This opinion paper proposes Sake as a model matrix to highlight bacterial role in traditional beverages that are considered exclusively mold/yeast-based. In addition, the opinion underlines the importance of considering all microbial determinants for a complete safety assessment.

## Bacterial Dna in Sake

Numerous microorganisms are present at the beginning of the sake production process because sake brewing is not performed under completely sterile conditions. The ethanol concentration increases with the growth of sake yeast. Although all (or most) microorganisms die due to the high ethanol concentration, their DNA is present in the final sake product. Thus, the DNA sequences in sake can be analyzed to determine and compare the microbial flora contained in sake (Bokulich et al., 2014; Koyanagi et al., 2016; Terasaki et al., 2017; Tsuji et al., 2018).

The bacterial DNA composition of sake is entirely different from that of the water used in sake production (Terasaki et al., 2018). That is, the bacterial DNA detected in sake is not derived from the water but rather from bacteria that have entered and probably briefly grown during the sake production process. Some lactic acid bacteria can survive at ethanol concentrations of 20%, and they cause spoiling when they contaminate sake (Suzuki et al., 2008). We have detected a high rate of lactic acid bacteria DNA sequences in spoiled sakes. Thus, DNA sequence comparison can be used as a powerful tool to analyze bacterial flora composition in sake. However, we must bear in mind that the detection of bacterial DNA in sake does not necessarily indicate that these bacteria are still alive.

## Bacterial Flora in *Moto*

Different sakes generally comprise different chemical components (Akaike et al., 2020), and the diversity of chemical components in sake is lower than that of the bacterial flora (Akaike et al., 2020; Terasaki and Nishida, 2020). Specific bacteria appear to affect specific chemical components during the production of the fermentation starter *moto*. For example, the ornithine composition increases and the arginine composition decreases during the production process of the fermentation starter *yamahai-moto* (also known as *kimoto*; traditional fermentation starter), and ornithine-producing *Latilactobacillus sakei* has been isolated from *yamahai-moto* (Tsuji et al., 2018). The bacteriocins produced by *Lactococcus lactis* inhibit the growth of sake-spoiling bacteria (Taniguchi et al., 2010). Although *Bacillus* sp. and *Staphylococcus* sp. predominate the early stage of *kimoto* production, lactobacilli predominate the late stage (Bokulich et al., 2014). Additionally, bacterial flora composition differs between the fermentation starters *yamahai-moto* and *sokujo-moto* (Terasaki et al., 2017).

These findings suggest that bacterial flora in sake may influence the chemical components and quality of sake products. Thus, bacteria that have inhabited a sake brewery for a long time could enter the sake brewing process and may be related to the bacterial flora and the characteristics of that sake.

## Bacterial Isolation From *Hatsuzoe*

The fermentation starter *moto* is mixed with *koji*, steamed rice, and water, and this is generally a three-step process, including *Hatsuzoe* (the first step), *Nakazoe* (the second step), and *Tomezoe* (the third step). Because bacteria cannot be isolated from sake products, we isolated bacteria from samples of the first mixture (*Hatsuzoe*). There are many live bacteria in *Hatsuzoe* (the first mixture of *moto* and *koji*) that can be identified and classified based on their 16S rDNA sequences. We obtained 46 isolates from six *Hatsuzoe* (the first mixture of *moto* and *koji*) samples from Brewery Toyama 1 (Terasaki and Nishida, 2020). Of them, 23, 12, 6, 2, 2, and 1 belonged to the bacterial genera *Kocuria, Staphylococcus, Bacillus, Leifsonia, Microbacterium*, and *Enterococcus*, respectively (Terasaki and Nishida, 2020). The detection of DNA associable to bacteria belonging to these genera underlines the importance, among the future perspectives, of a complete microbial safety assessment in sake productions that includes also prokaryotic organisms. In fact, some of these taxonomic units encompass species/strains of interest for food fermentations, but also undesired species/strains in the food chains (e.g., Tamang et al., 2017; Hanchi et al., 2018; Wu et al., 2019).

We isolated *Kocuria* sp. from all six *Hatsuzoe* (the first mixture of *moto* and *koji*) samples from Brewery Toyama 1 (Terasaki and Nishida, 2020). We also analyzed 16S rDNA sequencing results from 44 clear sake samples, three cloudy sake samples, and 11 *sake-kasu* (sake lee) samples from 33 sake breweries. *Kocuria* DNA was only detected in two samples of cloudy sake and one of *sake-kasu*, all of which were from Brewery Toyama 1 (Terasaki and Nishida, 2020). Interestingly, *Kocuria* DNA was not detected in any clear sakes. Our findings indicate that *Kocuria* DNA was not present in the sake solution because *Kocuria* cells are difficult to lyse by ethanol (Fujita et al., 2006). Thus, the *Kocuria* isolates inhabit Brewery Toyama 1 (Terasaki and Nishida, 2020) and are *kuratsuki* (= living in a sake brewery) bacteria from the sake brewery.

## *Kuratsuki* Bacteria and Yeast

In the past, different sake breweries used different yeast strains that inhabited their brewery, known as *kuratsuki* yeast. Today, sake brewing using *kuratsuki* yeast is rare. Many sake breweries buy and use selected sake yeasts that are managed by the Brewery Society of Japan (*Jozo-kyokai*). However, these yeasts were once established in selected sake breweries.

To our knowledge, no *kuratsuki* bacteria other than lactic acid bacteria had been reported prior to the publication of our paper (Terasaki and Nishida, 2020). Because it was thought that bacteria do not have a positive effect on the sake brewing process and cause negative effects, such as spoilage, *kuratsuki* bacteria have not been investigated. All or most *kuratsuki* yeasts are different strains of *Saccharomyces cerevisiae*. On the other hand, *kuratsuki* bacteria may differ at the genus or species level because *Kocuria* DNA was not detected in any other sake breweries except for Brewery Toyama 1 (Terasaki and Nishida, 2020). Thus, we hypothesize that *kuratsuki* bacteria have different functions in the sake production process in different sake breweries. We have sequenced the genomic DNA of the *Kocuria* isolates and are currently analyzing them. We believe that *kuratsuki* bacteria affect the sake quality, resulting in tastes and flavors that are specific to the brewery producing the sake.

*Staphylococcus* sp. and *Bacillus* sp. were isolated from 4/6 and 3/6 *Hatsuzoe* (the first mixture of *moto* and *koji*) samples, respectively, from Brewery Toyama 1 (Terasaki and Nishida, 2020), suggesting that these isolates may also be *kuratsuki* bacteria. Furthermore, *Staphylococcus* sp. and *Bacillus* sp. DNA were detected more frequently in sake than *Kocuria* DNA (Terasaki and Nishida, 2020). Thus, these DNAs are not specific to bacterial DNA in the sakes of Brewery Toyama 1 (Terasaki and Nishida, 2020) at the genus level. However, if the *Staphylococcus* sp. and *Bacillus* sp. isolates are specific to the sake brewery, their genomic sequences could differ at the species or strain level in each genus.

## Future Plan

The *hiire* pasteurization process halts the activities of enzymes of *koji* mold and sake yeast during the sake production process. Although enzymes, such as amylase and alcohol dehydrogenase, are essential for sake brewing, sake cannot be produced using only rice and these enzymes. Microorganisms are essential for sake production because they produce flavor and taste and influence the sake quality. Although bacteria die at the final stage of sake production, recent studies have shown that bacteria in sake are alive and grow temporarily during the sake production process. Thus, in the production process of sake, a bacterium interacts with another bacterium and sake yeast. *Kuratsuki* bacteria may play a key role in the interaction among microorganisms and may influence the sake quality. We are studying the functions of *kuratsuki* bacteria during sake brewing using comparative genome analyses and co-culturing experiments with these bacteria and sake yeast. We plan to change the characteristics of sake and produce a unique sake with different tastes and flavors by exchanging the *kuratsuki* bacteria from different sake breweries during the production process. Interest in sake is increasing around the world. Some Sake products are Geographical Indications (GIs) (https://www.nta.go.jp/publication/pamph/sake/04.pdf). In effect, an increasing interest is recognized to autochthonous microorganisms in the production of fermented traditional products (Capozzi and Spano, 2011; Capozzi et al., 2012, 2020). This opinion paper claims a possible role for autochthonous bacteria isolated from the sake productions among the factors included in the product specifications. At the same time, it highlights the importance of a complete microbial safety assessment that involves also bacteria.

## Author Contributions

HN wrote the manuscript.

## Conflict of Interest

The author declares that the research was conducted in the absence of any commercial or financial relationships that could be construed as a potential conflict of interest.
